# Additional sectioning and AI‐assisted diagnosis reveal underdiagnosis of serous (pre)malignancies in fallopian tube specimen from *BRCA*1/2 carriers

**DOI:** 10.1111/his.70249

**Published:** 2026-08-12

**Authors:** Anouk B Bouwmeester, Tamar A Gootzen, Marielle Nobbenhuis, Vilius Rudaitis, Caroline B van den Berg, Heleen J van Beekhuizen, Luthy SM Alcalá, Dineke PM Smedts, Nicole CM Visser, Viola Verhoef, Trudy GN Jonges, Ronald P Zweemer, Alicia Leon‐Catillo, Carlijn Lems, Joanne A de Hullu, Jeroen AWM van der Laak, Miranda P Steenbeek, Michiel Simons

**Affiliations:** ^1^ Department of Pathology Radboudumc Nijmegen the Netherlands; ^2^ Department of Gynecology and Obstetrics Radboudumc Nijmegen the Netherlands; ^3^ Department of Gynecological Oncology The Royal Marsden NHS Foundation Trust London UK; ^4^ Faculty of Medicine, Clinic of Obstetrics and Gynecology Vilnius University Vilnius Lithuania; ^5^ Department of Gynecology and Obstetrics ErasmusMC Rotterdam the Netherlands; ^6^ Department of Pathology Amphia hospital Breda the Netherlands; ^7^ Department of Gynecology and Obstetrics Amphia hospital Breda the Netherlands; ^8^ Department of Pathology PAMM Eindhoven the Netherlands; ^9^ Department of Gynecology and Obstetrics Maxima Medical Center Veldhoven the Netherlands; ^10^ Department of Pathology University Medical Center Utrecht Utrecht the Netherlands; ^11^ Department of Gynecological Oncology University Medical Center Utrecht Utrecht the Netherlands; ^12^ Department of Pathology Antoni van Leeuwenhoek Amsterdam the Netherlands; ^13^ Department of Gynecology and Obstetrics Catharina Hospital Eindhoven the Netherlands

**Keywords:** high‐grade serous carcinoma, ovarian cancer, salpingo‐oophorectomy, sampling error, serous tubal intraepithelial carcinoma

## Abstract

**Aim:**

Germline *BRCA1/2* pathogenic variant carriers are at increased risk for high‐grade serous carcinoma (HGSC) and are therefore advised to have a risk‐reducing salpingo‐oophorectomy (RRSO) around the age of 40. A risk of 0.9% to develop peritoneal HGSC (pHGSC) remains, which increases up to 27.5% when serous tubal intraepithelial carcinoma (STIC) is detected at RRSO. The relationship between the detection of STIC and the occurrence of pHGSC is still poorly understood. Here, we investigated the role of tissue sampling by examining deeper sections of the tubal tissue of RRSO specimens.

**Methods:**

Four groups of patients were included: (1) STIC without pHGSC (*n* = 5); (2) STIC with pHGSC (*n* = 4); (3) no STIC and no pHGSC (*n* = 5); and (4) no STIC and pHGSC (*n* = 5). Follow‐up time was 10 years or until the development of pHGSC. Deeper sections were cut at 150 μm intervals. A deep learning model was used to support STIC detection.

**Results:**

A focus of isolated STIC or HGSC was found in the deeper sections of all patients who developed pHGSC, while these patients did not have STIC or HGSC at the initial diagnosis. No patients with STIC and pHGSC showed HGSC in the deeper sections. We observed that five STICs presented as serous tubal intraepithelial lesions (STILs) in adjacent slides based on low Ki‐67 expression.

**Conclusions:**

Our findings underscore the hypothesis that pHGSC originates in the fallopian tube and confirm that invasiveness is not necessary for disease progression of STIC towards pHGSC. Furthermore, deeper sections might be of additional value when STIL is diagnosed.

## Graphical abstract

This paper shows that deeper sections reveal additional STICs and HGSCs in risk‐reducing salpingo‐oophorectomy specimens of patients who did not have a STIC or HGSC diagnosis but did develop peritoneal HGSC 10 years after surgery. This adds to the premise that HGSC originates in the fallopian tube.

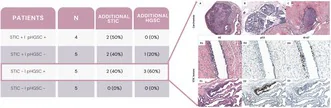

**Abbreviations**
DL: deep learning
H&E: haematoxylin and eosin
HGSC: high‐grade serous carcinoma
IHC: immunohistochemical
OC: ovarian carcinoma
PALGA: Pathologisch‐Anatomisch Landelijk Geautomatiseerd Archief
PV: pathogenic variant
RRSO: risk‐reducing salpingo‐oophorectomy
SEE‐FIM: Sectioning and Extensively Examining the FIMbriated end
STIC: serous tubal intraepithelial carcinoma

## Introduction

Ovarian carcinoma (OC) is the fifth most lethal cancer among women.^1^ As screening is not effective in decreasing mortality and symptoms tend to present at late‐stage disease, most patients have a poor prognosis.^1^, ^2^, ^3^ The general population has a lifetime risk of 1.3% of developing OC.^4^ Women with a *BRCA1* or *BRCA2* germline pathogenic variant (PV) have an increased risk, ranging from 17 to 48%.^5^, ^6^ As a result, they are recommended to undergo a risk‐reducing salpingo‐oophorectomy (RRSO) around the age of 40.^7^ Although RRSO is an effective preventive measure, a risk of developing high‐grade serous carcinoma (HGSC) in the peritoneal cavity remains (pHGSC).^8^

HGSC accounts for 70% of all OCs in general and 90% of OCs in *BRCA1/2* PV carriers specifically.^9^, ^10^, ^11^ Accumulating evidence points towards serous tubal intraepithelial carcinoma (STIC) as the precursor of HGSC, which has shifted focus towards the fallopian tubes as the primary site of origin, rather than the ovaries.^12^, ^13^, ^14^ In line with this hypothesis, a recent study has shown that the risk of developing pHGSC after RRSO increases by up to 27.5% when STIC is found during pathological examination. This risk is only 0.9% when no STIC is detected.^8^ So far, there is no consensus on the treatment or follow‐up of patients with STIC.^15^

An important factor in STIC follow‐up and treatment is accurate diagnosis. To increase detection of STIC, the Sectioning and Extensively Examining the FIMbriated end (SEE‐FIM) protocol was developed. This approach involves embedding the entire fallopian tube and creating longitudinal sections through the fimbriated end^16^ (Figure 1A). However, since STIC varies in size from 0.1 to 8 mm (median: 1.5 mm), and pathologists examine the fallopian tube at 2–3 mm increments, STIC may be overlooked.^8^ Additionally, misdiagnosis may occur if STIC turns out to be invasive deeper in the paraffin block, which may not be detected in the initial tissue sections. In both cases, sampling error would lead to misdiagnosis.

**Figure 1:**
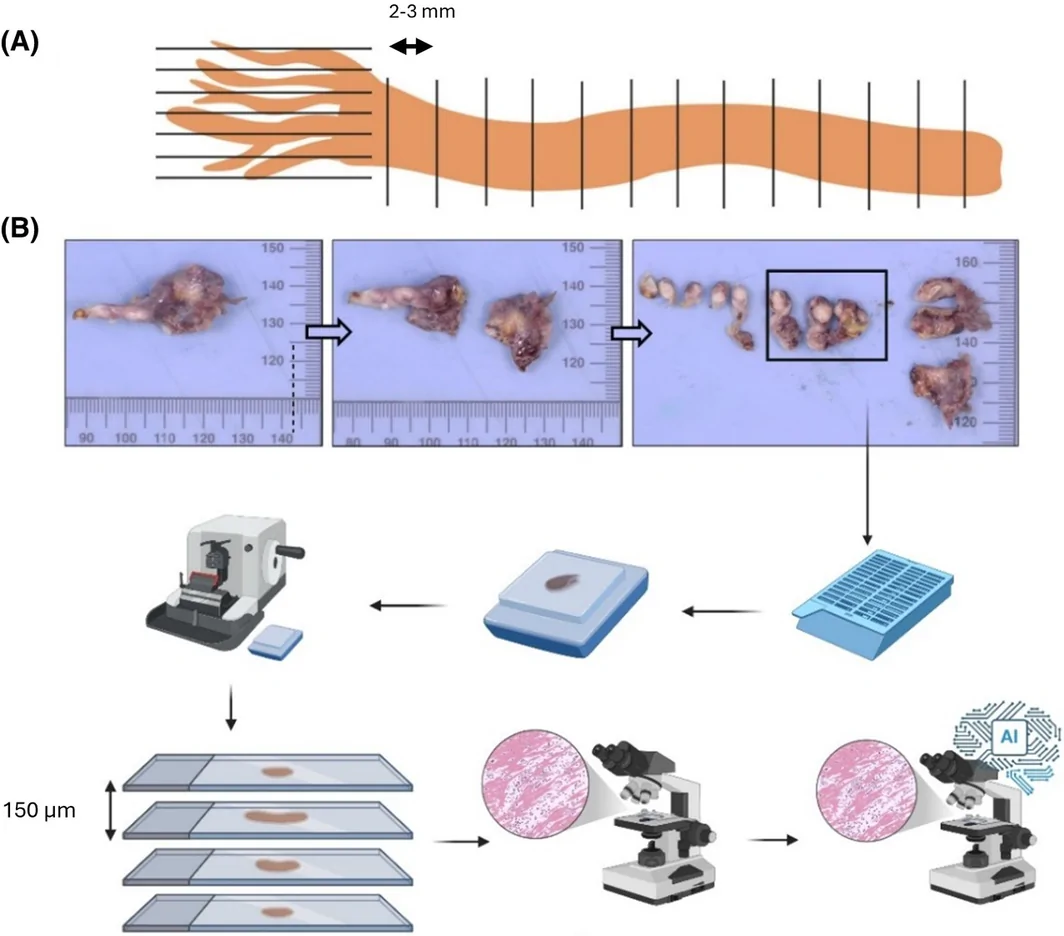
Workflow **(A)** Schematic representation of grossing according to the SEE‐FIM protocol. **(B)** Macroscopic pictures of a fallopian tube grossed according to the SEE‐FIM protocol and schematic representation of the workflow: deeper sections made at every 150 μm, unassisted morphological assessment, morphological assessment assisted by a deep learning model. Immunohistochemistry was performed based on morphological assessment.

Another challenge in STIC diagnosis is interobserver variability. When STIC was first identified, kappa scores for diagnosing STIC ranged from 0.33 to 0.39.^17^, ^18^ Reproducibility has improved with the adoption of diagnostic guidelines based on morphology and p53 and Ki‐67 immunohistochemistry.^19^ More recently, a deep learning (DL) algorithm was developed to assist pathologists in identifying STIC. In a reader study, this algorithm demonstrated an increase in sensitivity for STIC detection from 82% to 93%, and specificity rose from 92% to 97% when comparing assisted and unassisted reads.^19^

Not for all patients with pHGSC, STIC is detected in the fallopian tubes. One explanation is that STIC is missed because of sampling error. Another explanation is the concept of precursor escape, in which precursor lesions with a *TP53* mutation but lacking criteria of STIC (serous tubal intraepithelial lesion (STIL) and p53 signatures) could exfoliate into the peritoneal cavity.^20^ In the present study, we aimed to determine the effect of sampling error on the relationship between detection of STIC and pHGSC occurrence.

## Methods

### Study Design

For this retrospective pathology pilot study, patients were identified through previous publications,^21^, ^22^ via clinical contacts, a local search in the Dutch Pathologisch‐Anatomisch Landelijk Geautomatiseerd Archief (PALGA) at Radboudumc and a national PALGA search (2024‐84‐A1).^23^

Four groups of *BRCA1/2* PV carriers were included:

1. Women with isolated STIC at RRSO and no pHGSC after 10 years of follow‐up;
2. Women with isolated STIC at RRSO who developed pHGSC in the follow‐up period;
3. Women without STIC/HGSC at RRSO and no pHGSC after 10 years follow‐up; and
4. Women without STIC/HGSC at RRSO who developed pHGSC in the follow‐up period.

Exclusion criteria were: no approval for the use of data or residual tissue for research purposes, incompletely embedded fallopian tubes, and no availability of all fallopian tube tissue blocks. Ethical approval was obtained locally at Radboudumc (file number 2024‐1705). Patients were not informed about the findings in the deeper sections, as these did not have clinical consequences.

Original slides of the fallopian tubes were revised by a pathology resident and a gynaecologic pathologist (AB/MSi) independently. A third independent pathologist was consulted when no consensus was reached. Immunohistochemical (IHC) staining of Ki‐67 and p53 was additionally performed when results of these markers were not available. The criteria as proposed by Visvanathan *et al*. were followed for diagnosis.^17^ Morphology was decisive when the lesion was not present in the additional sections for IHC staining with Ki‐67 or p53.

### Data Collection

Cases were included from eight collaborating hospitals. Clinical data was collected from the electronic patient file and pseudonymized. Collected data included age at RRSO, type of *BRCA* PV, diagnosis of breast carcinoma, family history of OC, the RRSO pathology report and execution of concurrent hysterectomy. If applicable, previous therapy and the date of pHGSC diagnosis were collected. We reported the use of the SEE‐FIM protocol, IHC staining and the performance of deeper sections.

### Processing and Evaluation of Original Slides and Deeper Sections

Deeper sections were cut every 150 μm until there was no additional tissue remained in the block. Slides were stained with haematoxylin and eosin (H&E) and digitized into whole slide images (WSI; with a pixel size of 0.25 μm) using a Pannoramic P1000 scanner (3DHistech, Hungary). Multiple blank slides per case were saved for storage. The original slides were also digitized into WSIs. All WSIs were processed by a previously developed DL algorithm.^24^ For the present study, a STIC detection threshold of 0.3 on the DL algorithm likelihood output was applied, equal to the original study in which the model was validated.^19^ Assessment of all sections was performed in two steps: first, a pathology resident (AB) reviewed the WSI for the presence of STIC or HGSC. Subsequently, the DL algorithm was run which yielded bounding box annotations over the WSI. All DL detections were visually checked as the DL algorithm sometimes provides detections which are evidently false, for example, in areas with artefacts, morphologically normal loose cells and transitional epithelium (Figure 1).

For all slides containing suspicious areas with atypical epithelium, a serial section was stained with Ki‐67 and p53 immunohistochemistry (IHC). When this area was present in multiple consecutive sections of the same tissue block, only the first and last slides were IHC‐stained.

The morphology and immunophenotype were assessed independently by an experienced gynecologic pathologist (MSi) and a pathology resident (AB). They scored the tissue as normal/reactive epithelium, p53 signature, serous tubal intraepithelial lesion (STIL) or STIC based on the criteria of Visvanathan *et al*.^17^ If needed, consensus was reached by discussion. The number and size of STIC and/or HGSC foci were assessed for each tissue block. Size was expressed as the number of sections in which the lesion was present and the diameter measured on the slide.

### H&E and Immunohistochemical Staining

For immunohistochemical and H&E staining, blank 4 μm sections were cut from formalin‐fixed paraffin‐embedded blocks. For H&E staining a semi‐automatic stainer was used (Tissue‐Tek®PrismaTM, Sakura, Ultimo 74604). For immunohistochemical staining, deparaffinization, antigen retrieval (Envision Flex Tris High PH, 30 min at 97°C) and blocking with peroxidase‐blocking agent (Envision FLEX kit, Dako) were performed. Subsequently, slides were automatically incubated with p53‐antibody (clone DO‐7, Dako Omnis RTU) and Ki‐67 antibody (clone MIB‐1, Dako Omnis RTU). The slides, incubated with p53 antibody, were also incubated with Envision Flex Mouse Linker for 10 minutes at room temperature afterwards. Horseradish peroxidase (Envision FLEX kit, Dako) was used for all immunohistochemical stainings, and visualization was performed using di‐amino‐benzidine (DAB) + chromogen (Envision FLEX kit, Dako). Nuclear counterstaining was performed with haematoxylin. Staining was performed with a semi‐automatic stainer (PT link PT200, Dako, Ultimo A7941).

## Results

### Patient Characteristics

A total of 19 women were included: four women in the STIC+/pHGSC− group and five in the STIC+/pHGSC+, STIC−/pHGSC− and STIC−/pHGSC+ groups. Median age at RRSO was 47 years (interquartile range: 41–61). There were 13 *BRCA2 PV* carriers (68%) and 6 *BRCA1* PV carriers (42%). A peritoneal washing was performed at RRSO in 12/19 (63.2%) cases, showing normal cytology (Table 1). The use of SEE‐FIM was generally not specified in the pathology report.

**Table 1:** Baseline characteristics

	Group 1 STIC+/pHGSC– *N* = 4, *N* (%), median (range)	Group 2 STIC+/pHGSC+ *N* = 5, *N* (%), median (range)	Group 3 STIC−/pHGS– *N* = 5, *N* (%), median (range)	Group 4 STIC−/pHGS+ *N* = 5, *N* (%), median (range)
*Personal characteristics*				
Age at RRSO	55.5 (46–71)	61 (41–65)	40 (38–55)	47 (39–60)
Menopausal status				
Premenopausal	1 (25)	2 (40)	5 (100)	4 (80)
Postmenopausal	3 (75)	3 (60)	0 (0)	1 (20)
Pathogenic variant				
BRCA1	2 (50)	3 (60)	3 (60)	5 (100)
BRCA2	2 (50)	2 (40)	2 (40)	0 (0)
Breast cancer in medical history	4 (100)	3 (60)	2 (40)	1 (20)
Age at breast cancer	47.5 (36–80)	46 (40–48)	39^a^	31^b^
Tamoxifen usage	2 (50)	1 (33)	0 (0)	0 (0)
Family history of ovarian cancer				
Yes	1 (25)	1 (20)	0 (0)	3 (60)
No	2 (50)	2 (40)	1 (20)	1 (20)
Unknown	1 (25)	2 (40)	4 (80)	1 (20)
Living status				
Alive	4 (100)	3 (60)	5 (100)	3 (60)
Death	0 (0)	2 (40)	0 (0)	2 (40)
*Additional diagnostics during RRSO*				
Peritioneal washing				
Normal	4 (100)	3 (60)	2 (40)	3 (60)
Abnormal	0 (0)	0 (0)	0 (0)	0 (0)
Not performed/unknown	0 (0)	2 (40)	3 (60)	2 (40)

^a^ Two identical values; therefore, a range is not applicable.

^b^ Only one value present, therefore a range is not applicable.

### Pathologic Review of the Original Slides

All cases with an original STIC diagnosis exhibited unequivocal morphology and a p53 aberrant overexpression pattern. Ki‐67 positivity ranged between 10% and 95%. P53 immunohistochemistry was not performed in one case from the STIC+/pHGSC+ group (case number 6, Table 2). However, clonality analysis was conducted on this STIC and metachronous pHGSC which showed a clonal relationship based on a *TP53* mutation. In another case in the STIC+/pHGSC− group (case number 1), Ki‐67 IHC was not evaluated because the lesion was depleted in deeper sections.

**Table 2:** Characteristics of STIC and HGSC

Case	Original slides							Deeper sections					
	Diagnosis	Ki‐67 (%)	Number of foci	Diameter on slide (mm)	Number of sections	Location	Laterality	Diagnosis	Number of foci	Diameter on slide (mm)	Number of sections	Location	Laterality
*STIC+/pHGSC−*													
1	STIC	‐	1	0.8	4	Fimbriae	Unilateral	STIC	1	1.06	2	Fimbriae	Unilateral
2	STIC	67	1	4.69	1	Fimbriae	Unilateral	STIC	2	6.02; 1.68	2	Fimbriae	Unilateral
3	STIC	60	1	3.08	1	Fimbriae	Unilateral	STIC	1	2.99	6	Fimbriae	Unilateral
4	STIC	10; 40	2	1.26; 3.44	2	Fimbriae	Bilateral	STIC+HGSC and STIC+STIL	5	1.83	10	Fimbriae	Bilateral
*STIC+/pHGSC+*													
5	STIC	10	1	1.90	4	Lumen	Unilateral	STIC+STIL^a^	2	1.83	6	Lumen	Unilateral
6	STIC	30^b^	8	1.91	1	Fimbriae	Unilateral	STIC	14	1.65	14	Fimbriae and lumen	Bilateral
7	STIC	40	1	1.66	1	Fimbriae	Unilateral	STIC+STIL	2	1.10; 0.96	7	Fimbriae	Unilateral
8	STIC	>10	4	3.02	1	Fimbriae	Unilateral	STIC	8	1.94	12	Fimbriae and lumen	Unilateral
9	STIC	95	1	1.95	1	Fimbriae	Unilateral	STIC	1	1.79	4	Fimbriae	Unilateral
*STIC−/pHGSC+*													
10	P53 signature	NA	2	0.28; 0.35		Lumen	Unilateral	STIC+STIL	1	1.56	6	Fimbriae	Unilateral
11	‐							STIC	1	0.91	2	Fimbriae	Unilateral
12	‐							Exfoliated HGSC cells	1	1.15	1	Lumen	Unilateral
13	‐							HGSC	1	1.55	3	Fimbriae	Unilateral
14	‐							HGSC + STIC	1	4.29	9	Fimbriae	Unilateral
*STIC−/pHGSC−*													
15													
16													
17													
18													
19													

When more than two foci are present, the median of the diameter on the slide is given. For the diagnosis in the deeper sections column only STIC and HGSC are mentioned. STIC+STIL means one of the edges of the STIC was diagnosed as STIL.

^a^ Two times STIC+STIL, one original, one new.

^b^ Molecular analysis performed.

When reviewing previously performed sections, we found that three additional deeper sections were already analysed in two STIC cases, one from the STIC+/pHGSC− (case number 1) and one from the STIC+/pHGSC+ group (case number 5). One additional STIC focus with a p53‐negative aberrant pattern was detected in the STIC+/pHGSC– group in the original slides by the DL algorithm (case number 4) (Table 3).

**Table 3:** The detection methods of slides with STIC and STIL

		DL alone	DL and pathologic review	Pathologic review alone
STIC and STIL	Original slides	1	12	2
	Deeper sections	5	53	26

The number of slides with STIC or STIL present detected by each method. At the original diagnosis, nine STICs were diagnosed. In total, six additional sections were performed at the original diagnosis, of which five could be scanned. Three of these deeper sections were detected by DL. One additional focus on a different slide was detected by DL alone in a patient with a STIC diagnosis based on another slide.

When evaluating STIC characteristics from all patients with detected STICs in the original slides, we observed that STICs were located in the distal fimbriae in 8/9 (89%) patients and in the tubal lumen in 1/9 (11%) patients. The number of foci ranged between 1 and 8. One of the STIC−/pHGSC+ cases had a p53 signature (Table 2).

### Pathologic Review of the Deeper Sections

A median of 6 (range 2–12) tissue blocks per patient contained fallopian tube tissue, resulting in a total of 1,437 deeper sections. The median number of sections per tissue block was 12 (range 5–19), and the median number of deeper sections per patient was 60 (range 24–149). IHC was performed on 78 slides. Six discrepancies were noted between the pathologist and the resident in the classification of lesions based on morphology and IHC, which were resolved after discussion.

Previously undetected HGSC or STIC were present in the deeper sections in all (5/5, 100%) cases of the STIC−/pHGSC+ group (Figure 2). In 2/5 (40%) cases, STIC was found in the deeper sections without HGSC. In the three (60%) other cases, previously undetected HGSC was observed. Presentation of HGSC varied in these cases. The first case showed atypical exfoliated cells, which, due to the large amount and architectural complexity of cells, were interpreted as HGSC. The second case showed a combination of HGSC and STIC, and the last case showed only HGSC. In the last two described cases, a separate STIL was also found.

**Figure 2:**
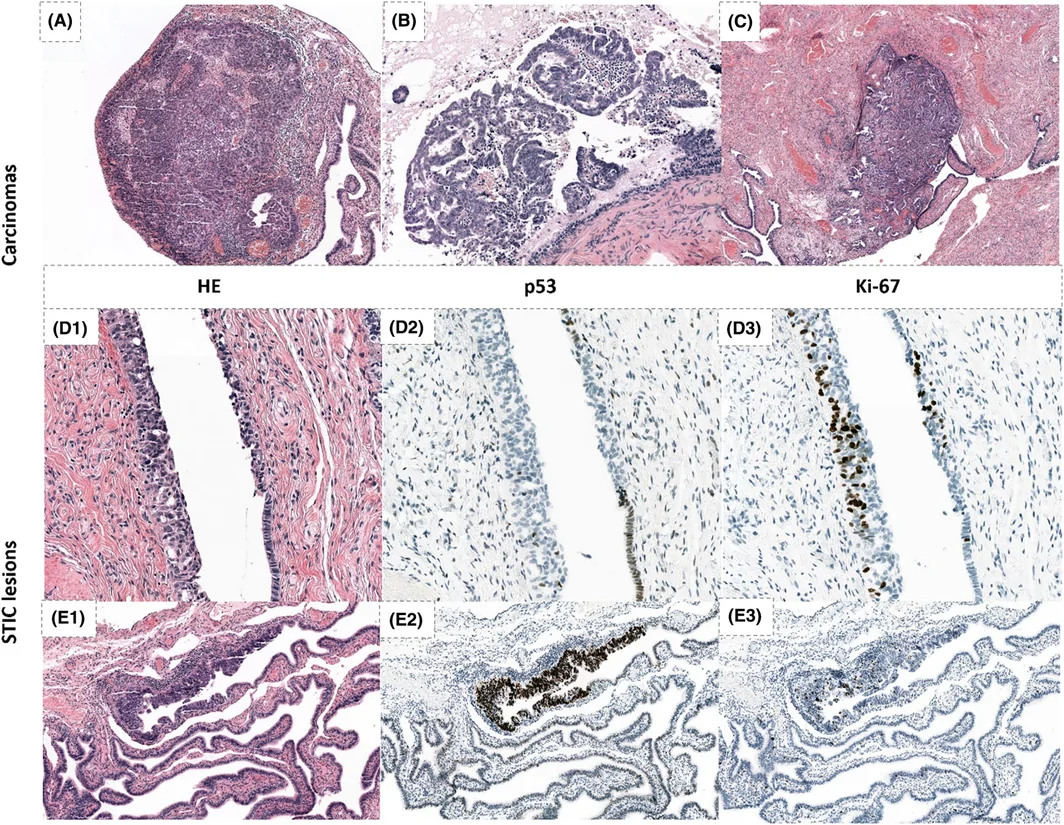
Lesions present in the STIC−/pHGSC+ group. Each letter indicates an individual case. **(A)** H&E staining of HGSC without STIC at 20× magnification. **(B)** H&E staining of exfoliated HGSC cells at 10× magnification considered diagnostic of HGSC. **(C)** H&E staining of HGSC with STIC at 5× magnification. **(D)** From left to right: H&E staining showing unequivocal morphology, p53 IHC showing a negative mutational staining pattern, Ki‐67 IHC showing >10% positivity. Pictures are at 20× magnification. **(E)** From left to right: H&E staining showing unequivocal morphology, p53 IHC showing a positive mutational staining pattern, Ki‐67 showing >10% positivity. Pictures are at 20× magnification.

In the STIC+/pHGSC− group, HGSC was found in the deeper sections in one case (Figure 3). In contrast, no carcinoma was found in the deeper sections in the STIC+/pHGSC+ group (Table 2). In both these groups, additional STICs and STILs were found. In the STIC−/pHGSC− group, no additional lesions were found.

**Figure 3:**
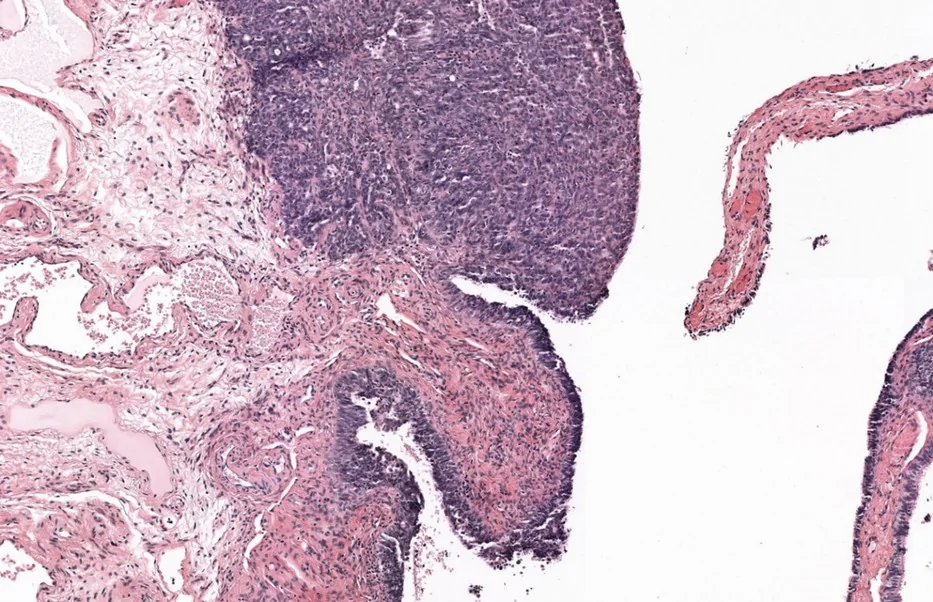
STIC accompanied by HGSC in a STIC+/pHGSC− patient. 10× magnification. [Colour figure can be viewed at wileyonlinelibrary.com]

We compared isolated STICs found in the original sections or deeper sections that occurred in pHGSC+ and pHGSC− patients. Multifocality was present in 4/7 (pHGSC+) versus 1/3 cases (pHGSC−) and the median number of sections with STIC was 8 (range 2–15) (pHGSC+) versus 6 (range 3–7) (pHGSC−). The diameter on the slide ranged between 0.24 and 6.30 mm (pHGSC+) versus 0.8 and 6.02 mm (pHGSC−). Isolated STIC was present in the tubal lumen in 3/7 (pHGSC+) versus 0/3 cases (pHGSC−). In 2/3 cases with luminal presence of STIC, the STIC was also multifocally present in the fimbriae.

In 5/20 (25%) STICs, one edge of the lesion was diagnosed as STIC, while the opposite edge was diagnosed as STIL based on a low Ki‐67 labelling index (Figure 4).

**Figure 4:**
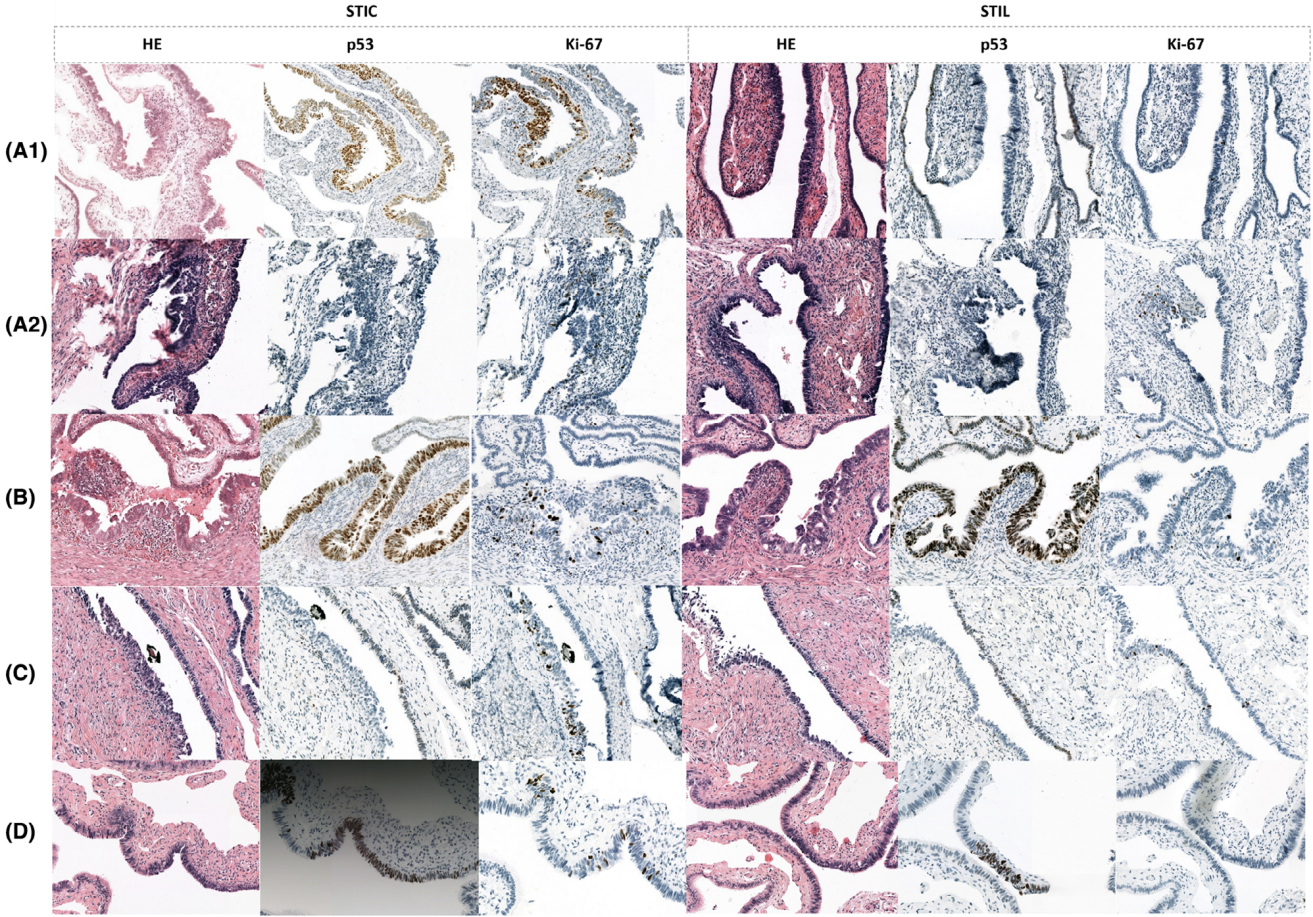
STIC and STIL classification in the same lesion. Each letter indicates an individual case and is depicted at 20× magnification. All the images show one border that met the criteria of STIC and the other border that met the criteria of STIL. All H&E staining's showed unequivocal morphology. P53 IHC showed positive mutational staining in **(B, D)** and negative mutational staining in **(C, A2)**. In **(A1)** both positive and negative mutational staining is seen in the lesion, with a positive mutational staining pattern on the STIC end and a negative mutational staining pattern on the STIL end. All cases show >10% Ki‐67 positivity in the STIC border of the lesion (left Ki‐67 column) and <10% Ki‐67 positivity in the STIL border of the lesion (right Ki‐67 column). [Colour figure can be viewed at wileyonlinelibrary.com]

The time to pHGSC ranged between 45 and 55 months for (exfoliated) HGSC, 54–90 months for unifocal isolated STIC and 32–75 months for multifocal isolated STIC.

## Discussion

This study shows that sampling error led to potential underdiagnosis of STIC and HGSC in patients who develop pHGSC. Furthermore, it confirms that invasive growth is not necessary for disease progression, as six patients only had STIC and no HGSC in the whole paraffin block and developed pHGSC. Lastly, we found that in 20% of STICs, one of the borders of the lesion met the diagnostic criteria of STIL.

Previous studies on sampling error only performed two to six deeper sections per tissue block, while we had a median of 12 deeper sections per tissue block. Mahe *et al*. investigated sampling effects in women without a detected STIC at RRSO who subsequently developed HGSC. They used two deeper sections at a 100 μm interval. Additional areas of STIC were identified in 4/56 (7.1%) cases,^25^ while we identified additional areas of STIC or HGSC in 5/5 (100%) cases. Furthermore, two other studies investigated sampling effects in the whole group of women undergoing RRSO. Visvanathan *et al*. performed three serial sections on all fallopian tube tissue blocks; in a subset, they reviewed an additional three sections from the other side of the tissue blocks that contained the fimbriated end. In this last subset, the prevalence of HGSC/STIC/STIL increased from 6.3% to 11.9%, indicating that doubling the number of sections doubled the number of detected lesions.^26^ However, Rabban *et al*. showed complete concordance between detection of STIC and tubal carcinoma in the original sections and the additional three multilevel sections of fimbriae blocks. None of the STICs showed invasion in the additional sections in this study, in contrast to our findings.^27^ Since in the whole RRSO population the vast majority of patients would fall in the STIC−/pHGSC− group, systematically performing deeper sections in all RRSO specimen is likely not cost‐effective. Furthermore, since the use of SEE‐FIM is not specifically mentioned in pathology reports, our results might overestimate the value of deeper sections.

An interesting finding in the present study is that one of the borders of the lesion was diagnosed as STIC while the other border was diagnosed as STIL because the Ki‐67 labelling index did not reach the 10% threshold in 5/20 (20%). For clinical practice, this sparks the discussion whether Ki‐67 should be omitted when morphology is unequivocal and p53 is aberrant, as Perrone *et al*. recommend.^28^ Furthermore, in a Delphi study among 34 expert gynaecological pathologists, consensus on the mandatory use of Ki‐67 staining was not reached.^29^ However, the latest edition of the WHO Female Genital Tumours states that Ki‐67 needs to be >10% in order to diagnose STIC, based on the criteria of Visvanathan.^17^, ^30^ Ultimately, the use of IHC improves reproducibility among pathologists, but there are currently no studies available correlating staining patterns with clinical outcomes. Furthermore, our results indicate that deeper sections could be helpful in clinical practice for lesions diagnosed as STIL, but that fall short of current STIC criteria.

We observed a possible trend when comparing isolated STICs from patients with pHGSC+ and pHGSC–. STICs in pHGSC+ women seem to be present in both the fimbriae and the lumen, with more foci and in a higher number of sections. However, the numbers are very small. Therefore, international collaboration to register patients with STIC and collect their pathological and clinical information is necessary to improve future research on the predictive value of STIC morphology and immunohistochemistry (e.g. NCT06393543).

This is the first study to investigate sampling effects with a division into four groups: STIC +/− and pHGSC +/− women. The main strength of this study is that we used 150 μm intervals throughout the complete fallopian tube specimen. This provided a more extensive approach to determining the rate of underdiagnosis than the previous literature.

Limitations of this study include the fact that the deep learning model detected fewer STICs/STILs than expected based on prior validation of this model. It should be noted that the model was not trained to detect carcinomas, which will be the focus of future extensions. As this study was not designed to validate a DL model, performance measures could not be calculated. Differences in performance are most probably caused by a lack of generalization of the model. Furthermore, the resident was not blinded to the outcome. Therefore, a bias in selecting the cases for immunohistochemistry could be present. Lastly, this study has a small sample size as it is a pilot study, impacting the generalizability of the data. However, since STIC is rare and the available tissue is therefore limited, this pilot study was effective in signalling trends and generating new hypotheses.

## Conclusion

Our findings show that sampling effects contribute to the underdiagnosis of STIC and HGSC in patients who develop pHGSC without an initial STIC diagnosis at RRSO. Furthermore, we confirmed that invasion is not necessary for disease progression to pHGSC. Lastly, our results suggest that deeper sections might be of additional value when STIL is diagnosed based on low ki‐67 expression.

## Author contributions statement

Conception: AB, TG, JH, JL, MSt, MSi; Data collection: AB, TG, MN, VR, CB, HB, LA, HS, NV, VV, GJ, RZ, AL, MSt, MSi; AI‐related tasks: CL; Data analysis: AB, TG, MSi; Writing: TG, AB, JH, JL, MSi, MSt. All authors approved the final version of the manuscript.

## Funding information

This study was funded by the Dutch Cancer Society (KWF) (project number 12950).

## Conflicts of interest

JL was a member of the advisory boards of Philips, the Netherlands and ContextVision, Sweden and received research funding from Philips, the Netherlands, ContextVision, Sweden and Sectra, Sweden in the last 5 years. He is the chief scientific officer (CSO) and shareholder of Aiosyn BV, the Netherlands. AL received honoraria, travel, accommodation and expenses from GlaxoSmithKline. The other authors have no conflicts of interest to declare.

## Data Availability

The data that support the findings of this study are available from the corresponding author upon reasonable request.
